# Enhanced efficiency and endothelial protection of the intraocular lens shell technique in phacoemulsification: a retrospective stratified analysis by nuclear hardness

**DOI:** 10.3389/fmed.2026.1910499

**Published:** 2026-09-23

**Authors:** Huizhen Wang, Haibo Deng, Guizhen Pan, Liyuan Zhou

**Affiliations:** Department of Ophthalmology, Yingtan People’s Hospital, Yingtan, Jiangxi, China

**Keywords:** corneal endothelial cell loss, cumulative dissipated energy, intraocular lens shell technique, nuclear hardness, phacoemulsification, posterior capsule rupture

## Abstract

**Background:**

The intraocular lens (IOL)-shell technique may improve intraoperative stability during phacoemulsification, but whether its benefits vary by nuclear hardness remains uncertain. This study evaluated the safety and efficiency of the IOL-shell technique and explored its effects across nuclear hardness subgroups.

**Methods:**

This retrospective study included 200 patients undergoing phacoemulsification: 100 treated with the IOL-shell technique and 100 treated with the conventional technique. Outcomes included operative time, cumulative dissipated energy (CDE), corneal endothelial cell loss, posterior capsule rupture (PCR), corneal edema, and best-corrected visual acuity (BCVA) at 1 month. Subgroup and interaction analyses were performed according to nuclear hardness (≥ Grade IV vs. ≤ Grade III).

**Results:**

Baseline characteristics were comparable between groups. Compared with the conventional group, the IOL-shell group had shorter operative time (12.79 ± 2.00 vs. 15.13 ± 2.52 min, *P* < 0.001), lower CDE (8.36 ± 2.05 vs. 12.03 ± 2.78 units, *P* < 0.001), less endothelial cell loss (10.74 ± 3.55% vs. 14.59% ± 3.95%, *P* < 0.001), a lower corneal edema rate (18.0% vs. 32.0%, *P* = 0.022), and better 1-month BCVA (0.29 ± 0.08 vs. 0.36 ± 0.12 logMAR, *P* < 0.001). PCR was numerically lower with the IOL-shell technique but was not statistically significant overall (2.0% vs. 8.0%, *P* = 0.101). In eyes with nuclear hardness ≥ Grade IV, the IOL-shell technique was associated with shorter operative time, lower CDE, reduced endothelial cell loss, and lower PCR and corneal edema rates. Interaction analysis showed a significant technique-by-nuclear hardness interaction for operative time (*β* = −1.58, 95% *CI*: −2.93 to −0.23; *P* for interaction = 0.022), but not for other outcomes.

**Conclusions:**

The IOL-shell technique improved surgical efficiency, reduced ultrasound energy use, enhanced endothelial protection, decreased corneal edema, and promoted early visual recovery after phacoemulsification. Its efficiency advantage was particularly evident in eyes with nuclear hardness ≥ Grade IV. The apparent reduction in PCR should be interpreted cautiously because of the limited number of events.

## 1 Introduction

Cataract remains one of the leading causes of visual impairment worldwide, and phacoemulsification with intraocular lens (IOL) implantation is currently the standard surgical treatment for visually significant cataract (1, 2). With advances in surgical devices and techniques, cataract surgery has become increasingly safe and efficient (3, 4). However, complex cataract cases, particularly those with high nuclear hardness, continue to pose important intraoperative challenges (5, 6). Dense nuclear cataracts often require greater ultrasound energy, longer surgical manipulation, and more intensive fluidics, which may increase the risk of corneal endothelial injury, postoperative corneal edema, and intraoperative complications such as posterior capsule rupture (PCR) (7, 8).

Corneal endothelial cells play a crucial role in maintaining corneal transparency by regulating stromal hydration (9). Because human corneal endothelial cells have limited regenerative capacity, excessive endothelial cell loss after cataract surgery may lead to persistent corneal edema and delayed visual recovery (10, 11). Several intraoperative factors, including cumulative dissipated energy (CDE), operative time, fluid turbulence, and mechanical trauma, have been associated with endothelial damage during phacoemulsification. Therefore, surgical strategies that reduce ultrasound energy exposure and improve intraoperative stability are particularly important in eyes with dense nuclei or other high-risk features.

The intraocular lens-shell technique is a modified phacoemulsification approach in which the IOL is implanted before removal of the final nuclear fragment (12). In this technique, the implanted IOL may serve as a protective mechanical barrier between the residual nuclear material and the posterior capsule, while also helping to stabilize the capsular bag and maintain intraocular spatial configuration during the final phase of surgery. These theoretical advantages suggest that the IOL-shell technique may reduce posterior capsule movement, improve control of nuclear fragments, decrease ultrasound energy use, and protect the corneal endothelium.

Although the IOL-shell technique has been reported as a potentially useful approach in dense cataract surgery, evidence regarding its clinical effectiveness remains limited (11). In particular, it is still unclear whether the benefits of this technique are uniform across different degrees of nuclear hardness or more evident in eyes with harder nuclei. Most previous studies have focused on overall comparisons between surgical techniques, while stratified analyses by nuclear hardness and formal interaction assessments have been insufficiently explored. Clarifying this issue may help identify patients who are most likely to benefit from the IOL-shell technique and support more individualized surgical decision-making.

Therefore, this study aimed to evaluate the safety and efficiency of the IOL-shell technique in patients undergoing phacoemulsification, with particular attention to nuclear hardness. We compared intraoperative parameters, postoperative complications, endothelial cell loss, and early visual outcomes between the IOL-shell and conventional techniques. In addition, subgroup and interaction analyses were performed to determine whether the effect of the IOL-shell technique differed according to nuclear hardness.

## 2 Methods

### 2.1 Study design and patients

This retrospective study was conducted at the Department of Ophthalmology, Yingtan People's Hospital, Yingtan, Jiangxi, China, and included 200 consecutive patients who underwent phacoemulsification cataract surgery. Patients were assigned to the conventional or IOL-shell group according to the surgical technique performed during the study period. The conventional group included 100 patients who underwent standard phacoemulsification during the earlier study period, whereas the IOL-shell group included 100 patients who underwent phacoemulsification using the IOL-shell technique after this approach had been adopted in routine clinical practice.

Patients were eligible for inclusion if they met the following criteria: (1) age ≥ 50 years; (2) diagnosis of cataract requiring phacoemulsification with intraocular lens implantation; (3) nuclear hardness Grade III–V according to the Emery-Little classification; and (4) available clinical, surgical, and postoperative follow-up data.

Patients were excluded if they had (1) a history of previous ocular surgery; (2) severe corneal disease or pre-existing corneal endothelial decompensation; (3) ocular trauma; (4) active ocular inflammation or infection; (5) ocular diseases that could independently affect postoperative visual acuity, including diabetic retinopathy, age-related macular degeneration, macular edema, retinal vascular diseases, uveitis, glaucoma with visual field impairment, or other significant retinal pathology; or (6) incomplete clinical or surgical data.

The study protocol was reviewed and approved by the Medical Ethics Committee of Yingtan People's Hospital (approval date: May 6, 2025). As this was a retrospective study based on routinely collected clinical data, the requirement for written informed consent was waived by the ethics committee.

### 2.2 Surgical techniques

All procedures in both groups were performed by the same senior cataract surgeon with 15 years of experience in phacoemulsification and an annual surgical volume of approximately 600 cataract surgeries. The same phacoemulsification platform, identical machine parameter settings, ophthalmic viscosurgical device, and foldable posterior chamber intraocular lens were used whenever appropriate according to the standardized surgical protocol, thereby minimizing variability related to surgical operators and equipment. In the conventional group, phacoemulsification and aspiration of the lens nucleus and cortex were completed before implantation of a foldable posterior chamber intraocular lens into the capsular bag.

After approximately 80%–90% of the lens nucleus had been emulsified, leaving only the final nuclear fragment (approximately 10%–20% of the original nuclear volume), the foldable posterior chamber IOL was implanted into the capsular bag. The remaining nuclear fragment was then emulsified and aspirated with the implanted IOL serving as a protective mechanical barrier between the residual nucleus and the posterior capsule.

### 2.3 Nuclear hardness assessment and subgroup definition

Nuclear hardness was assessed preoperatively by slit-lamp examination and graded according to the Emery-Little classification (Grades I–V). All preoperative grading was performed by the same experienced cataract surgeon to ensure consistency of assessment. For stratified analyses, patients were divided into two subgroups: eyes with nuclear hardness ≥ Grade IV and eyes with nuclear hardness ≤ Grade III. Because the inclusion criteria were limited to complex cataracts with nuclear hardness Grade III–V, the ≤ Grade III subgroup consisted of Grade III nuclei only and did not include Grade I or II soft nuclear cataracts.

This subgroup classification was selected because eyes with nuclear hardness ≥ Grade IV are generally considered surgically more challenging and may require greater ultrasound energy and longer manipulation during phacoemulsification.

Although the IOL-shell technique was originally proposed for dense nuclear cataracts, after its adoption into routine clinical practice at our institution it was applied as the standard surgical approach for eligible complex cataract cases with Grade III–V nuclear hardness rather than being selectively reserved only for dense nuclei. Therefore, the ≤ Grade III subgroup in the present study consisted exclusively of Grade III nuclei, and stratified analyses were performed to determine whether the effectiveness of the technique differed between Grade III and Grade IV–V cataracts.

### 2.4 Data collection

Demographic and clinical data were collected from medical records, including age, sex, diabetes status, preoperative best-corrected visual acuity (BCVA), and nuclear hardness grade. Surgical data included operative time and cumulative dissipated energy (CDE). Postoperative data included posterior capsule rupture (PCR), corneal edema, corneal endothelial cell loss, and BCVA at 1 month after surgery.

BCVA was recorded using the logarithm of the minimum angle of resolution (logMAR) scale. Preoperative endothelial cell density was collected as a baseline ocular parameter and included in the baseline comparison. Corneal endothelial cell density was measured preoperatively and again at the routine 1-month postoperative follow-up visit using the same non-contact specular microscope (Topcon SP-3000P, Topcon Corporation, Japan), which coincided with the assessment of BCVA. Endothelial cell loss was calculated as the percentage reduction from the preoperative endothelial cell density to the value measured at the 1-month postoperative examination. Posterior capsule rupture (PCR) was identified from intraoperative surgical records. Corneal edema was assessed by slit-lamp examination on postoperative day 1 and recorded as a binary outcome (present or absent) based on the presence of clinically visible corneal stromal edema. Because of the retrospective nature of the study, the postoperative examiners were not masked to the surgical technique.

### 2.5 Outcome measures

The primary intraoperative outcomes were operative time and CDE. The main safety and postoperative outcomes included PCR, corneal edema, and corneal endothelial cell loss. BCVA at 1 month postoperatively was evaluated as an early visual outcome.

Subgroup outcomes were assessed separately in eyes with nuclear hardness ≥ grade IV and ≤ grade III. In addition, interaction analyses were performed to evaluate whether the association between surgical technique and outcomes differed according to nuclear hardness.

### 2.6 Statistical analysis

Continuous variables were expressed as mean ± standard deviation and compared between groups using independent-samples *t* tests. Categorical variables were expressed as number (percentage) and compared using chi-square tests or Fisher's exact tests, as appropriate.

Because only 10 PCR events occurred, multivariable logistic regression analysis for PCR was performed only as an exploratory, hypothesis-generating supplementary analysis. No ROC analysis was performed, and no prediction model was developed or validated. The model included surgical technique, nuclear hardness, age, and diabetes. Because PCR was a low-frequency event in this cohort, the multivariate logistic regression analysis was considered exploratory.

Subgroup analyses were conducted according to nuclear hardness category. For continuous outcomes, interaction effects between surgical technique and nuclear hardness were assessed using linear regression models including surgical technique, nuclear hardness, and their interaction term. For categorical outcomes, interaction effects were assessed using logistic regression models including the same interaction term.

To further assess the robustness of the main findings, additional multivariable regression analyses were performed for the main outcomes. Linear regression models were used for continuous outcomes, including operative time, CDE, endothelial cell loss, and 1-month BCVA. Logistic regression models were used for binary outcomes, including PCR and corneal edema. These models adjusted for age, nuclear hardness, diabetes, preoperative BCVA, and preoperative endothelial cell density. Anterior chamber depth was not included because it was not consistently available in the retrospective records. Propensity score matching was not performed because the sample size was modest and baseline characteristics were generally comparable between groups.

All statistical analyses were performed using R software. A two-sided *P* value < 0.05 was considered statistically significant.

## 3 Results

### 3.1 Baseline characteristics

The baseline characteristics of the study population are summarized in Table 1. A total of 200 patients were included, with 100 patients in the IOL-shell group and 100 in the conventional group. There were no significant differences between the two groups in age (70.31 ± 8.03 vs. 71.72 ± 8.61 years, *P* = 0.231), sex distribution (male: 48.0% vs. 50.0%, *P* = 0.777), preoperative BCVA (1.30 ± 0.28 vs. 1.32 ± 0.34 logMAR, *P* = 0.607), proportion of nuclear hardness ≥ Grade IV (67.0% vs. 69.0%, *P* = 0.762), or diabetes prevalence (25.0% vs. 27.0%, *P* = 0.747). Preoperative endothelial cell density was also comparable between the IOL-shell and conventional groups (2524.88 ± 204.11 vs. 2480.29 ± 163.56 cells/mm^2^, *P* = 0.090). These findings indicate that the two groups were well balanced at baseline.

**Table 1:** Baseline characteristics of the study population.

Variable	IOL-shell group (*n* = 100)	Conventional group (*n* = 100)	*P* value
Age (years)	70.31 ± 8.03	71.72 ± 8.61	0.231
Male, *n* (%)	48 (48.0%)	50 (50.0%)	0.777
Preoperative BCVA (logMAR)	1.30 ± 0.28	1.32 ± 0.34	0.607
Nuclear hardness ≥ Grade IV, *n* (%)	67 (67.0%)	69 (69.0%)	0.762
Diabetes, *n* (%)	25 (25.0%)	27 (27.0%)	0.747
Preoperative endothelial cell density (cells/mm^2^)	2524.88 ± 204.11	2480.29 ± 163.56	0.090

BCVA, best-corrected visual acuity; logMAR, logarithm of the minimum angle of resolution.

Continuous variables are presented as mean ± standard deviation; categorical variables are presented as number (percentage). Between-group comparisons were performed using independent-samples *t* tests for continuous variables and chi-square or Fisher's exact tests for categorical variables, as appropriate.

### 3.2 Intraoperative outcomes

The intraoperative outcomes are shown in Table 2. Compared with the conventional group, the IOL-shell group had a significantly shorter operative time (12.79 ± 2.00 vs. 15.13 ± 2.52 min, *P* < 0.001). In addition, cumulative dissipated energy (CDE) was significantly lower in the IOL-shell group than in the conventional group (8.36 ± 2.05 vs. 12.03 ± 2.78 units, *P* < 0.001). These findings suggest that the IOL-shell technique was associated with improved intraoperative efficiency and reduced ultrasound energy use.

**Table 2:** Intraoperative parameters.

Variable	IOL-shell group (*n* = 100)	Conventional group (*n* = 100)	*P* value
Operative time (min)	12.79 ± 2.00	15.13 ± 2.52	<0.001
CDE (units)	8.36 ± 2.05	12.03 ± 2.78	<0.001

CDE, cumulative dissipated energy.

Data are presented as mean ± standard deviation. Between-group comparisons were performed using independent-samples *t* tests.

### 3.3 Postoperative complications and clinical outcomes

Postoperative complications and clinical outcomes are summarized in Table 3 and illustrated in Figure 1. The incidence of posterior capsule rupture (PCR) was numerically lower in the IOL-shell group than in the conventional group, although the difference did not reach statistical significance (2.0% vs. 8.0%, *P* = 0.101). Because only 10 PCR events occurred, the PCR-related findings were considered exploratory and hypothesis-generating.

**Figure 1:**
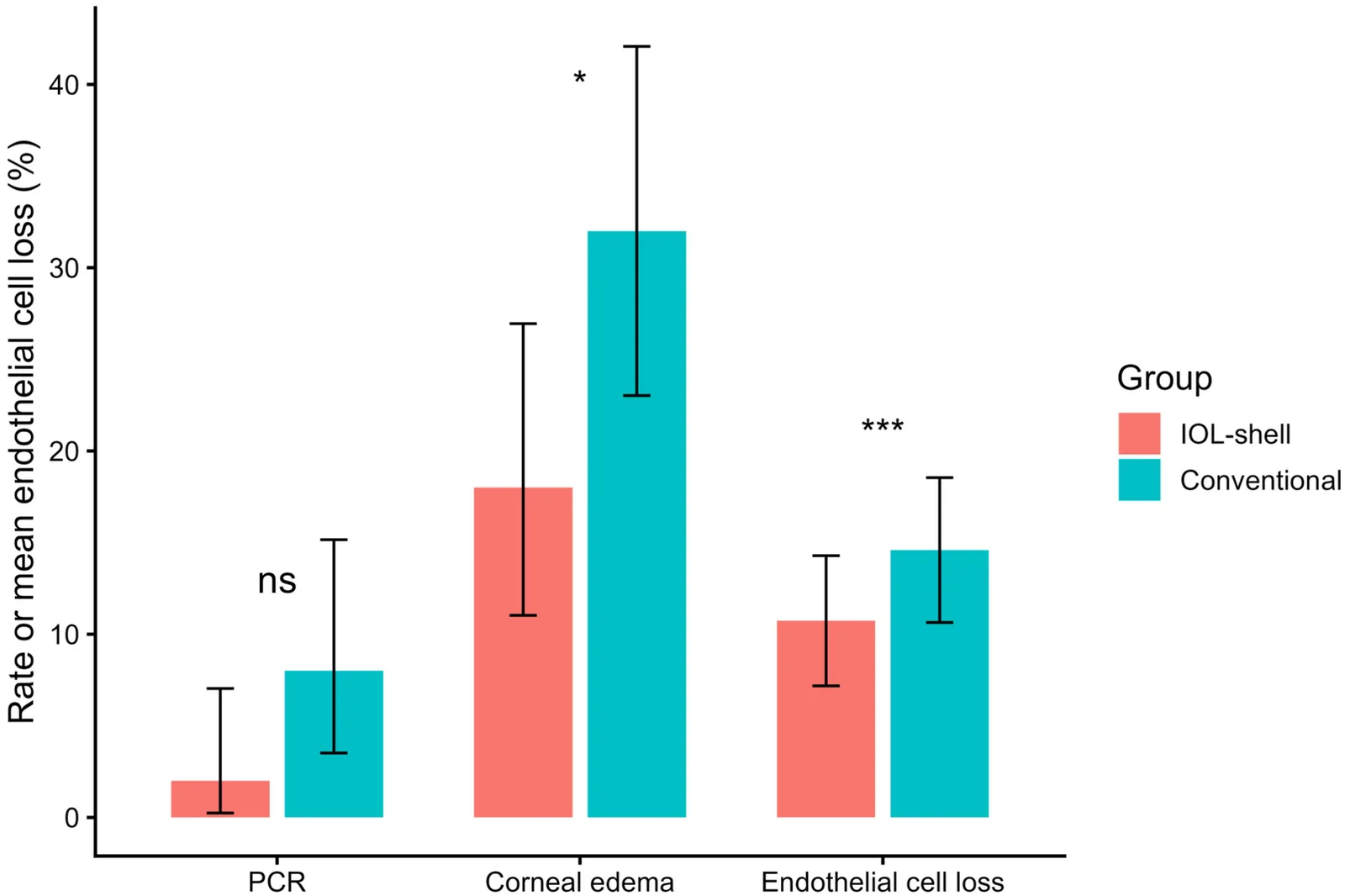
Comparison of postoperative complications and endothelial cell loss.

**Table 3:** Postoperative outcomes and complications.

Variable	IOL-shell group (*n* = 100)	Conventional group (*n* = 100)	*P* value
PCR, *n* (%)	2 (2.0%)	8 (8.0%)	0.101
Corneal edema, *n* (%)	18 (18.0%)	32 (32.0%)	0.022
Endothelial cell loss (%)	10.74 ± 3.55	14.59 ± 3.95	<0.001
BCVA at 1 month (logMAR)	0.29 ± 0.08	0.36 ± 0.12	<0.001

BCVA, best-corrected visual acuity; PCR, posterior capsule rupture; logMAR, logarithm of the minimum angle of resolution.

Continuous variables are presented as mean ± standard deviation; categorical variables are presented as number (percentage). Between-group comparisons were performed using independent-samples *t* tests for continuous variables and chi-square or Fisher's exact tests for categorical variables, as appropriate.

The rate of corneal edema was significantly lower in the IOL-shell group compared with the conventional group (18.0% vs. 32.0%, *P* = 0.022). In addition, the IOL-shell group showed significantly less endothelial cell loss than the conventional group (10.74 ± 3.55% vs. 14.59 ± 3.95%, *P* < 0.001). Visual outcomes at 1 month postoperatively were also better in the IOL-shell group, as reflected by lower BCVA values on the logMAR scale (0.29 ± 0.08 vs. 0.36 ± 0.12, *P* < 0.001). The endothelial cell density measurements were obtained at the same 1-month postoperative visit.

Overall, these findings indicate that the IOL-shell technique was associated with reduced corneal edema, better endothelial protection, and improved early postoperative visual recovery.

### 3.4 Subgroup analysis stratified by nuclear hardness

Subgroup analyses stratified by nuclear hardness are presented in Table 4. In eyes with nuclear hardness ≥ Grade IV, the IOL-shell group showed significantly shorter operative time than the conventional group (12.51 ± 2.16 vs. 15.36 ± 2.45 min, *P* < 0.001). CDE was also significantly lower in the IOL-shell group (8.49 ± 2.23 vs. 12.10 ± 2.47 units, *P* < 0.001). In addition, endothelial cell loss was significantly reduced in the IOL-shell group compared with the conventional group (10.52 ± 3.07% vs. 14.89 ± 3.88%, *P* < 0.001).

**Table 4:** Subgroup analysis stratified by nuclear hardness.

Outcome	Nuclear hardness ≥ Grade IV			Nuclear hardness ≤ Grade III		
	Conventional (*n* = 69)	IOL-shell (*n* = 67)	*P value*	Conventional (*n* = 31)	IOL-shell (*n* = 33)	*P value*
Operative time (min)	15.36 ± 2.45	12.51 ± 2.16	<0.001	14.63 ± 2.65	13.36 ± 1.52	0.024
CDE (units)	12.10 ± 2.47	8.49 ± 2.23	<0.001	11.88 ± 3.42	8.09 ± 1.62	<0.001
Endothelial cell loss (%)	14.89 ± 3.88	10.52 ± 3.07	<0.001	13.93 ± 4.09	11.17 ± 4.39	0.011
PCR, *n* (%)	6 (8.7)	0 (0.0)	0.028	2 (6.5)	2 (6.1)	1.000
Corneal edema, *n* (%)	24 (34.8)	9 (13.4)	0.004	8 (25.8)	9 (27.3)	0.894

CDE, cumulative dissipated energy; PCR, posterior capsule rupture.

Continuous variables are presented as mean ± standard deviation; categorical variables are presented as number (percentage). Between-group comparisons within each nuclear hardness subgroup were performed using independent-samples *t* tests for continuous variables and chi-square or Fisher's exact tests for categorical variables, as appropriate.

For postoperative complications in the ≥ Grade IV subgroup, PCR occurred less frequently in the IOL-shell group than in the conventional group (0.0% vs. 8.7%, *P* = 0.028). The rate of corneal edema was also significantly lower in the IOL-shell group (13.4% vs. 34.8%, *P* = 0.004).

In eyes with nuclear hardness ≤ Grade III, the IOL-shell group also showed shorter operative time (13.36 ± 1.52 vs. 14.63 ± 2.65 min, *P* = 0.024), lower CDE (8.09 ± 1.62 vs. 11.88 ± 3.42 units, *P* < 0.001), and reduced endothelial cell loss (11.17 ± 4.39% vs. 13.93 ± 4.09%, *P* = 0.011). However, there were no statistically significant differences between the two groups in PCR (6.1% vs. 6.5%, *P* = 1.000) or corneal edema (27.3% vs. 25.8%, *P* = 0.894).

Overall, the IOL-shell technique was associated with improved intraoperative efficiency and endothelial protection across both nuclear hardness subgroups, with more evident safety-related benefits in eyes with nuclear hardness ≥ Grade IV.

### 3.5 Interaction analysis

Interaction analysis was performed to further evaluate whether the effect of the IOL-shell technique differed according to nuclear hardness. As shown in Table 5, a significant interaction between surgical technique and nuclear hardness was observed for operative time (*β* = −1.58, 95% *CI*: −2.93 to −0.23, *P* for interaction = 0.022). This finding indicates that the reduction in operative time associated with the IOL-shell technique was more pronounced in eyes with nuclear hardness ≥ Grade IV. The subgroup-specific operative times according to surgical technique and nuclear hardness are illustrated in Figure 2.

**Table 5:** Interaction analysis.

Outcome	β (interaction)	*95% CI*	*P* for interaction
Operative time	−1.58	(−2.93, −0.23)	0.022
CDE	−0.19	(−1.72, 1.34)	0.806
Endothelial cell loss	−1.61	(−3.86, 0.65)	0.16
PCR	—	—	0.993
Corneal edema	—	—	0.068

CDE, cumulative dissipated energy; *CI*, confidence interval; PCR, posterior capsule rupture.

Interaction effects for continuous outcomes were estimated using linear regression models including surgical technique, nuclear hardness, and their interaction term. Interaction effects for categorical outcomes were evaluated using logistic regression models.

**Figure 2:**
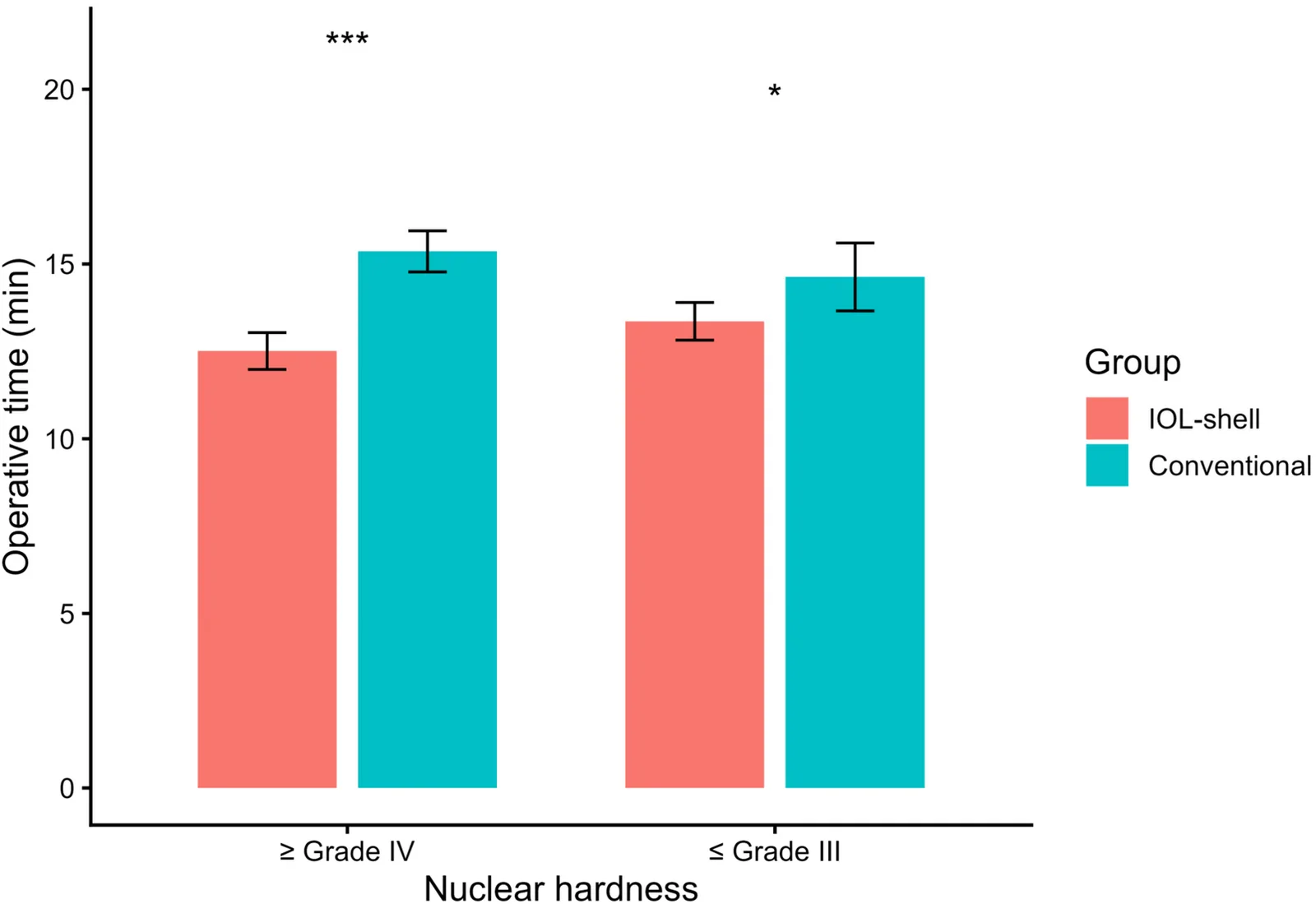
Operative time according to surgical technique stratified by nuclear hardness. Bars represent mean operative time, and error bars indicate 95% confidence intervals.

In contrast, no significant interaction effects were observed for CDE (*P* for interaction = 0.806), endothelial cell loss (*P* for interaction = 0.160), PCR (*P* for interaction = 0.993), or corneal edema (*P* for interaction = 0.068). These results suggest that, except for operative time, the effects of the IOL-shell technique on ultrasound energy use, endothelial protection, and postoperative complications were generally consistent across nuclear hardness subgroups.

### 3.6 Adjusted sensitivity analysis

Adjusted multivariable regression analyses were performed to assess the robustness of the main findings. After adjustment for age, nuclear hardness, diabetes, preoperative BCVA, and preoperative endothelial cell density, the IOL-shell technique remained significantly associated with shorter operative time, lower CDE, reduced endothelial cell loss, lower risk of corneal edema, and better 1-month BCVA. The association between the IOL-shell technique and PCR remained directionally protective but was not statistically significant, consistent with the primary analysis. These findings support the robustness of the main results.

## 4 Discussion

This retrospective comparison study of 200 patients who had undergone phacoemulsification surgery found that using the IOL-shell technique was linked to a number of benefits compared with the use of the traditional technique. Namely, patients from the IOL-shell group demonstrated significantly shorter operative time, decreased cumulative dissipated energy (CDE), fewer losses of the corneal endothelial cells, less corneal edema development, and superior BCVA outcomes one month after the operation. Despite being numerically smaller in the IOL-shell group, the overall incidence of PCR was not statistically different between the two groups under consideration. The positive effects of the IOL-shell technique were further observed in the subset of patients having nuclear hardness ≥ Grade IV, which included decreased operative time, CDE, losses of the corneal endothelial cells, incidence of PCR, and corneal edema development. Nevertheless, interaction analysis revealed that there were no interactions between nuclear hardness and technique regarding CDE, corneal endothelial cell loss, incidence of PCR, and corneal edema development.

Our findings are generally consistent with those reported by Luo et al., who first introduced the IOL-shell technique for dense nuclear cataracts (12). In their original study, implantation of the intraocular lens before removal of the final nuclear fragment improved intraoperative safety by protecting the posterior capsule and facilitating removal of the residual nucleus. Similarly, we observed that the IOL-shell technique was associated with lower cumulative dissipated energy, reduced corneal endothelial cell loss, less postoperative corneal edema, and improved early visual recovery. Furthermore, our subgroup analysis demonstrated that these advantages were particularly evident in eyes with nuclear hardness ≥ Grade IV, supporting the concept that the technique may be especially beneficial in more challenging cataract surgery.

One difference between our findings and those of Luo et al. is that we observed a significantly shorter operative time with the IOL-shell technique, whereas the original study reported no statistically significant difference. Several factors may explain this discrepancy. First, our cohort included a relatively high proportion of eyes with dense nuclear cataracts, in which improved stabilization of the capsular bag and residual nuclear fragments may facilitate more efficient emulsification and aspiration. Second, advances in phacoemulsification systems and accumulated surgical experience since the original report may have enhanced the procedural efficiency of the IOL-shell technique. Finally, differences in study design, patient characteristics, and operative time definitions may also have contributed to the observed discrepancy. Although the approximately 2-minute reduction in operative time may appear modest, it is clinically relevant in complex cataract surgery because even a small reduction in surgical duration may decrease cumulative ultrasound exposure, intraocular manipulation, and fluid turbulence, thereby contributing to better endothelial preservation and earlier postoperative recovery. This interpretation is also supported by our subgroup analysis, in which the reduction in operative time was significantly greater in eyes with nuclear hardness ≥ Grade IV, suggesting that the procedural efficiency of the IOL-shell technique becomes more apparent in technically demanding cataract surgery.

Our findings are consistent with recent evidence emphasizing the importance of reducing ultrasound energy and improving phacoemulsification efficiency in complex cataract surgery. In a 2025 study of hard nucleus cataracts, Sato reported that the eight-chop technique resulted in relatively low corneal endothelial cell density loss, and endothelial loss was significantly correlated with phaco time, CDE, and fluid volume, supporting the clinical relevance of minimizing intraoperative energy exposure in dense cataracts (6). Similarly, Ichikawa et al. evaluated phacoemulsification parameters across different nuclear hardness grades and found that CDE increased with nuclear hardness, while device- or technique-related differences in energy efficiency were more evident in harder lenses (13). A recent systematic review and meta-analysis comparing phaco-chop with divide-and-conquer also concluded that phaco-chop caused less corneal endothelial hazard and required less delivered ultrasound energy, further supporting the concept that techniques reducing intraocular energy exposure may improve endothelial safety (14). In addition, recent studies have shown that corneal endothelial cell loss after phacoemulsification is associated with factors such as CDE, total operating time, anterior chamber depth, lens thickness, and baseline endothelial status, and that early corneal edema is closely related to subsequent endothelial cell loss (15). Together, these findings are broadly consistent with our observation that the IOL-shell technique reduced CDE, endothelial cell loss, and corneal edema, especially in eyes with higher nuclear hardness.

Several mechanisms may explain the observed advantages of the IOL-shell technique. First, early implantation of the intraocular lens before removal of the final nuclear fragment may provide a mechanical barrier between the residual lens material and the posterior capsule, thereby stabilizing the capsular bag and reducing posterior capsule movement during the most vulnerable phase of surgery (16, 17). Second, the implanted IOL may help maintain intraocular spatial stability, reduce anterior chamber fluctuation, and improve control of nuclear fragments during phacoemulsification (18). Third, by improving surgical stability, the technique may allow more efficient emulsification and aspiration, thereby reducing operative time and CDE (13, 19). Since excessive ultrasound energy and fluid turbulence are important contributors to corneal endothelial injury, the reduction in CDE observed in our study provides a plausible explanation for the lower endothelial cell loss and corneal edema in the IOL-shell group (20, 21). The stronger effect observed in eyes with nuclear hardness ≥ Grade IV is also clinically reasonable, because dense cataracts require greater ultrasound energy, longer manipulation, and more stable intraoperative control.

Despite these potential advantages, the IOL-shell technique also has inherent technical limitations. Pre-implantation of the IOL may reduce the available working space in the anterior chamber and capsular bag, making subsequent manipulation of residual nuclear fragments more technically demanding. Adequate maintenance of layered ophthalmic viscosurgical device protection is therefore essential to preserve intraocular space and protect the corneal endothelium. In addition, if small residual nuclear fragments migrate behind the implanted IOL, they may be more difficult to visualize and remove, theoretically increasing the risk of posterior capsule traction or injury. Therefore, this technique requires careful surgical judgment, stable anterior chamber control, and sufficient surgeon experience, particularly in eyes with zonular weakness, small pupils, or unstable capsular support.

There are several limitations of this study. First, the retrospective, single-center design may limit the generalizability of the findings. Second, the two groups were distinguished based on the chronological order of treatment; therefore, residual confounding due to time effects, surgical experience, perioperative management, or device settings cannot be completely excluded. In addition, postoperative examiners were not masked to the surgical technique, which may have introduced assessment bias for clinically evaluated outcomes such as corneal edema. Third, although baseline characteristics were generally comparable between groups, some potentially relevant factors, including pupil size, zonular weakness, anterior chamber depth, and detailed nuclear morphology, were not fully adjusted for in the analysis. Another important limitation is that postoperative follow-up was limited to 1 month. Therefore, only early postoperative outcomes were evaluated, whereas clinically important long-term outcomes, such as the ease of posterior capsule polishing, posterior capsular opacification (PCO), postoperative refractive stability, and other late postoperative complications, were not assessed. Consequently, the short-term benefits observed in this study should not be interpreted as evidence of sustained long-term superiority of the IOL-shell technique. In addition, because only 10 PCR events occurred, the multivariable logistic regression analysis was considered exploratory, and no prediction model was developed or validated. Larger prospective, multicenter studies with longer follow-up are needed to confirm the present findings and to further evaluate both the long-term safety and effectiveness of the IOL-shell technique.

Overall, the IOL shell approach showed a shorter duration of operation, a smaller CDE, fewer lost endothelial cells, lesser corneal edema, and earlier recovery of visual acuity in patients who underwent phacoemulsification. The superior efficiency of the procedure was especially noted in those with nuclear hardness grade ≥ Grade IV. While the PCR was smaller overall and statistically less in the dense nucleus group, the small number of incidents requires further careful consideration.

The study results imply that the IOL shell approach may be considered an effective surgical technique in achieving better efficiency and endothelial cell preservation during complicated cataract surgery.

## Data Availability

The raw data supporting the conclusions of this article will be made available by the authors, without undue reservation.
