# Supplementary material for: Enhanced efficiency and endothelial protection of the intraocular lens shell technique in phacoemulsification: a retrospective stratified analysis by nuclear hardness
Source: Front Med (Lausanne). 2026 Sep 23;13:1910499. doi: 10.3389/fmed.2026.1910499 (PMC13641994; doi:10.3389/fmed.2026.1910499)
Supplement: Supplementary file 1 [file Supplementaryfile1.docx]

**Supplementary Table S1. Adjusted associations between surgical technique and main outcomes**

| **Outcome** | **Effect estimate for IOL-shell technique** | ***95% CI*** | ***P value*** |
| --- | --- | --- | --- |
| Operative time | *β* = −2.37 min | −3.02 to −1.73 | <0.001 |
| CDE | *β* = −3.73 units | −4.43 to −3.04 | <0.001 |
| Endothelial cell loss | *β* = −3.86% | −4.91 to −2.80 | <0.001 |
| BCVA at 1 month | *β* = −0.07 logMAR | −0.10 to −0.04 | <0.001 |
| Corneal edema | *OR* = 0.42 | 0.21 to 0.83 | 0.014 |
| PCR | *OR* = 0.21 | 0.04 to 1.06 | 0.058 |

**Note:** Models were adjusted for age, nuclear hardness, diabetes, preoperative BCVA, and preoperative endothelial cell density. Continuous outcomes were analyzed using linear regression, and binary outcomes were analyzed using logistic regression.

**Supplementary Table S2. Exploratory multivariable logistic regression analysis for PCR**

| **Variable** | ***OR*** | ***95% CI*** | ***P value*** |
| --- | --- | --- | --- |
| IOL-shell technique | 0.24 | 0.04–1.02 | 0.081 |
| Nuclear hardness ≥ Grade IV | 0.36 | 0.05–1.95 | 0.254 |
| Age | 1.02 | 0.95–1.10 | 0.576 |
| Diabetes | 3.48 | 0.61–19.88 | 0.163 |

**Note:** This analysis was exploratory and hypothesis-generating because only 10 PCR events occurred. No prediction model was developed or validated.
